# Effect of Ambient Lighting on Intraocular Pressure Rhythms in Rats

**DOI:** 10.1167/iovs.65.10.16

**Published:** 2024-08-08

**Authors:** Christina M. Nicou, Christopher L. Passaglia

**Affiliations:** 1 Medical Engineering Department, University of South Florida, Tampa, Florida, United States; 2 Ophthalmology Department, University of South Florida, Tampa, Florida, United States

**Keywords:** circadian rhythm, IOP telemetry, constant dark (DD), constant light (LL)

## Abstract

**Purpose:**

The purpose of this study was to determine the effects of ambient lighting on intraocular pressure (IOP) rhythmicity and variability.

**Methods:**

IOP was continuously recorded by wireless telemetry from rats under light/dark (LD), dark/light (DL), asymmetric (6L18D and 18D6L), constant dark (DD), and constant light (LL) cycles. In some DD experiments, 1-hour light pulses were presented at varying times. IOP rhythmicity and variability were respectively quantified via cosinor analysis and peak detection algorithms that identified transient and sustained fluctuations.

**Results:**

Rat IOP peaked at night and troughed during the day with LD amplitude of 8.7 ± 3.4 mm Hg. Rhythmicity persisted in DD and LL with a free-running period of 24.1 ± 0.3 and 25.2 ± 0.4 hours, respectively. Peak-to-trough amplitude was approximately 60% smaller in LL, often disappeared after 1 to 2 weeks as daytime IOP drifted 2.6 ± 1.5 mm Hg higher, and returned to approximately 60% larger in LD. Rhythmicity was similarly impacted but resynchronized to DL over 4 to 6 days. Rhythmicity was unaltered by short photoperiods (6L18D), but the nocturnal IOP elevation was markedly shortened by long photoperiods (18L6D) and temporarily lowered to daytime levels by light pulses during the subjective night. Transient and sustained event rate, amplitude, interval, and energy content were nearly identical in LD, DD, and LL.

**Conclusions:**

Aqueous humor dynamics of rat eyes are intrinsically configured to set IOP at daytime levels. Circadian clock input modulates these dynamics to elevate IOP at night. Light at night blocks this input, sending IOP back to daytime levels. Effects of abnormal lighting on IOP rhythmicity may contribute to pressure-related ocular neuropathies.

Most behavioral and physiological processes exhibit endogenous rhythms with a near 24-hour period in the absence of environmental cues. The rhythms are coordinated and controlled in mammals by a circadian clock located in the suprachiasmatic nucleus (SCN). The central clock receives light information from the eyes that allows it to entrain to the ambient light-dark cycle and anticipate day and night. In turn, the clock sends neural and humoral signals throughout the body that drive rhythms in some tissues and synchronize rhythms driven by peripheral clocks in other tissues.^1^^,^^2^ Circadian rhythm disorders due to impaired light perception or clock dysfunction underlie a myriad of cognitive and health problems.^3^^,^^4^

In the eye, a 24-hour oscillation in intraocular pressure (IOP) has been documented in a variety of mammals,^5^^–^^11^ including humans.^12^ A common finding is that IOP is lower during the day and higher at night in both nocturnal and diurnal animals (except birds^13^^,^^14^). The periodic pressure swings have been attributed to a circadian clock because they continue in constant darkness.^6^^–^^10^ The rhythm is important clinically as glaucoma is associated with ocular hypertension and IOP is typically measured during the day. The magnitude and breadth of IOP elevations go unmonitored at night, which is unfortunate because recent studies report that nocturnal spikes are predictive of disease progression.^15^^,^^16^

A challenge with circadian IOP research has been data collection. Round-the-clock tonometry is laborious and disrupts the subject's sleep-wake pattern, so even the most exhaustive studies take only a handful of IOP readings per day. The readings are variable and were often averaged across either days or subjects to decrease ocular and operator noise. The sparse data can cause misestimation of rhythm amplitude, period, and phase. It could also lead to misperception of arrhythmicity under constant lighting conditions because rhythm timing would drift without daily photoentrainment and cancel upon averaging over 24-hour intervals.^17^ Moreover, tonometry readings can be artificially elevated by stress responses from subject handling or restraint^18^^,^^19^ and lowered by light exposure when subjects awaken at night.^20^^–^^22^ IOP telemetry systems have thereby been developed to surmount these limitations. The systems have seen limited use, though, for investigating effects of ambient illumination on IOP beyond constant darkness.^23^^–^^25^ The objective of this study was to quantitatively characterize IOP rhythmicity and variability of rats in different lighting environments that probe circadian clock function.

## Methods

Experiments were conducted in compliance with the National Institutes of Health guide for the care and use of laboratory animals, ARVO Statement for the use of animals in ophthalmic and vision research, and protocols approved by the Institutional Animal Care and Use Committee at the University of South Florida. Brown-Norway rats (male retired breeders, 300–400 g) were purchased from a commercial vendor (Envigo, Indianapolis, IN, USA) and housed under a standard 12-hour light (6 AM–6 PM)/12-hour dark (6 PM–6 AM) cycle in a humidity- and temperature-controlled room (22°C) with food and water freely available.

### IOP Recording

After at least 1 week of room acclimation, a silicone microcannula (OD = 200 um and ID = 100 um; AS One International, Santa Clara, CA, USA) was implanted in the anterior chamber of one eye. The cannula was sutured to the sclera and fed to a custom skull-mounted coupler connected via plastic tubing to a laboratory-built wireless IOP sensor worn by the animal as a backpack. The cannula and connective tubing were filled with balanced salt solution, 3 mM moxifloxacin hydrochloride, 1.3 mM enoxaparin sodium, and 2.2 mM triamcinolone acetonide. Surgical procedures and sensor properties are detailed in prior publications.^19^^,^^26^ IOP data were collected at 0.25 hertz (Hz) and transmitted round-the-clock for weeks to months to a laptop. The sensor was calibrated by manometry before cannula implantation and no drift in calibration was noted upon explantation.

### Manipulation of Ambient Illumination

Implanted animals were entrained to the standard light/dark (LD) cycle for at least 1 week in a ventilated environmental control unit (ECU). In addition to minimizing external disturbances, the ECU (BIO-C36; Tecniplast, Buguggiate, Italy) allowed for independent manipulation of environmental lighting. ECU illuminance during light and dark phases was 250 and 0.002 lux, respectively. Animals were subsequently exposed for up to 2 weeks to a constant dark (DD), constant light (LL), reversed light/dark (DL), 18-hour light/6-hour dark (18L6D), or 6-hour light/18-hour dark (6L18D) cycle. The different lighting conditions probe rhythm periodicity (DD and LL), rhythm entrainment dynamics (DL), and rhythm sensitivity to photoperiod length (6L18D and 18L6D). Between conditions, animals were returned to LD for at least 1 week until re-entrained to the standard illumination cycle. In some DD experiments, a 1-hour light pulse was presented at select times of day spaced at least 2 days apart to probe clock control of IOP. Animal status inside the ECU was monitored with an infrared camera, and animal upkeep in DD was performed under dim red illumination.

### Identification of Transient and Sustained IOP Fluctuations

IOP fluctuations were parsed into transient and sustained events. Details of the event detection and parsing algorithm have been published.^27^ In short, events were identified using two parameters: peak prominence (amount a local maximum must exceed adjacent local minima to qualify as an event) and peak separation (minimum time interval between detected events). Peak prominence and peak separation were set at 1 mm Hg and 2 minutes, respectively, to curtail noise-triggered events. Prominent peaks separated more than this time interval were defined as transient events and subtracted from the IOP record. The algorithm was then reapplied to the residual record, and remaining peaks more than 20 minutes apart were defined as sustained events. They were subsequently subtracted from the residual record as well, leaving a smooth waveform comprised of a diurnal oscillation atop a constant IOP baseline. The biomechanical energy applied to outflow tissues was then calculated, as previously detailed,^27^ by integrating IOP variance over event duration and multiplying by the reported outflow resistance of rat eyes (43.5 min·mm Hg/µl^28^). This gives an approximation of applied energy because outflow resistance was not measured and may differ across animals.

### Data Analysis

IOP records were processed using MATLAB software (The Mathworks, Natick, MA, USA), and spurious data were removed with a combination of median and lowpass filters having a 28-s filter width.^19^ Rhythmicity was quantified by regressing LD, DD, LL, and DL data to a multicomponent cosinor function,^29^ given by:

$$P\left(t\right)=\bar{P}+\sum_{n}\tilde{P_{n}}\left(\frac{1+\cos\left(2\pi \left(t+\theta_{1}\right)n/T\right)}{2}\right),$$

where $\bar{P}$ is baseline IOP during subjective day, T is rhythm period, $\tilde{P_{n}}$ is peak-to-trough amplitude of the nth cosinor, and θ_*n*_ is phase of the nth cosinor (in hours with respect to midnight). Subjective day refers to the time of minimum IOP and subjective night to the time of maximum IOP, which might not align in the ECU with day and night in the outside world. Cosinor regression was performed on data collected at the very end of each lighting condition when rhythm responses to lighting changes had stabilized in waveform. Based on analysis of LD records of increasing length, it was determined that at least 3 days of data gave reliable estimates of rhythm parameters. Additional days were included in the analysis if the data were available and rhythm waveform was stable in order to maximize confidence in period and phase estimates, which are particularly sensitive to slow variations in IOP. For LL, cosinor regression was also performed on the first 3 days of data collected upon return to LD. Statistical comparisons were conducted using SigmaPlot software (Systat, San Jose, CA, USA) with significance assessed at α of 0.05. Data that passed a Shapiro-Wilk test are expressed as mean ± standard deviation, and group differences were evaluated using a paired Student's *t*-test or a 1-way repeated measures ANOVA with a Tukey test for multiple comparisons. Data that were found to be not normally distributed are summarized as median with lower and upper quartiles in brackets, and group differences were evaluated using a Mann-Whitney rank sum test or a 1-way repeated measures ANOVA on ranks with a Tukey test for multiple comparisons.

## Results

**Figure 1.**
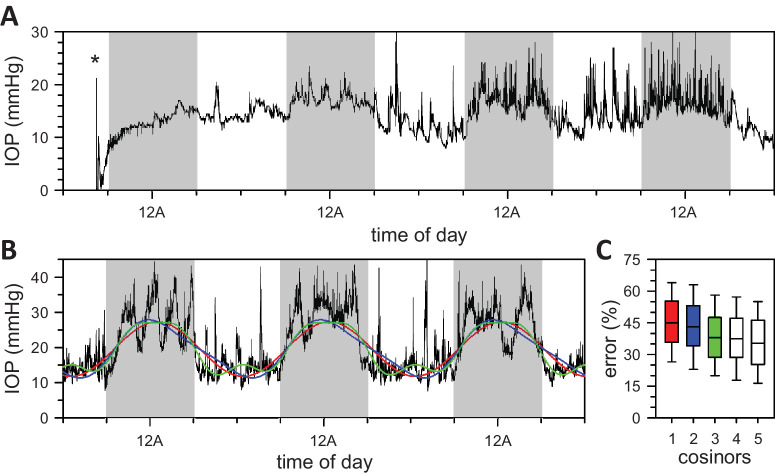
Rat IOP rhythm in LD. (**A**) After cannula implantation (*asterisk*), IOP could take a few days to resume cycling between low daytime levels and high nighttime levels. (**B**) Representative fits of a one- (*red*), two- (*blue*), and three-component (*green*) cosinor model to IOP data recorded over three LD cycles. (**C**) Distribution of fitting errors across all IOP recordings for several cosinor models (*n* = 15). Errors are expressed as the percent of unexplained variance relative to the total variance in IOP records. *Gray shading* indicates the dark phase of the LD cycle.

IOP was recorded round-the-clock for 52 ± 26 days in 15 rats. All animals exhibited a diurnal IOP rhythm that was entrained to the LD cycle before ambient lighting conditions were altered. The IOP rhythm was often weak or absent after eye surgery and could take a few LD cycles to recover. Figure 1A illustrates a post-surgical record in which IOP held fairly steady before endogenous rhythmicity resumed around 24 to 48 hours after cannula implantation. Rhythm features were quantified by multi-cosinor analysis. Figure 1B shows single, double, and triple cosinor fits to an LD record. The single cosinor can be seen to capture IOP rhythmicity, whereas the triple cosinor better describes the sudden flips between low daytime (light phase) and high nighttime (dark phases) IOP levels. Figure 1C shows that multi-cosinor analysis accounted for 30% to 70% of the total variance in IOP records, with the percentage error largely determined by the amplitude and frequency of slow IOP fluctuations across animals. Increasing the number of cosinors in the analysis produced better fits with progressively lower error, but the benefits were not statistically significant (*P* = 0.38). IOP rhythmicity was henceforth analyzed for different lighting conditions using the single cosinor model. Baseline IOP, peak-to-trough amplitude, period, and phase averaged 10.7 ± 2.3 mm Hg, 8.7 ± 3.4 mm Hg, 24.0 ± 0.2 hours, and 0.1 ± 0.6 hours, respectively, across animals in LD (*n* = 15). The Table lists the collection of ambient lighting conditions to which subsets of these animals were exposed.

**Table.** Sample Sizes for Each Ambient Lighting Condition

Lighting Condition^*^	Sample Size
DD	10
LL	7
DL	7
6L18D	3
18L6D	3
1-h pulses	3

* D, dark; L, light.

### Constant Dark Cycle

**Figure 2.**
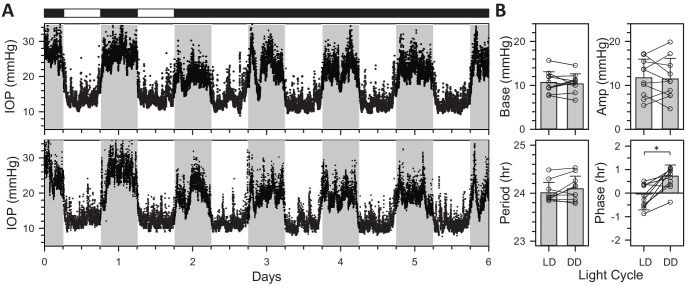
Effect of constant dark on IOP rhythmicity. (**A**) IOP data recorded concurrently from two rats that were entrained to LD and then exposed to DD. *White and black horizontal bars*, respectively, indicate light and dark periods of ECU illumination experienced by the animals. *Gray vertical bars* indicate the dark phase of the ongoing LD cycle outside the ECU. (**B**) Summary of rhythm properties in DD from cosinor analysis (*n* = 10). *Symbols* indicate baseline, amplitude, period, and phase estimates for individual animals. Error bars give standard deviation. *Asterisks* indicate statistically significant differences.

Figure 2A shows IOP data collected simultaneously from 2 rats placed for several days in DD. The IOP rhythm was prominent for both animals under LD and persisted in DD without disruption or deterioration, indicating that it is driven by an internal circadian clock and not by pupillary responses to light nor by light itself. Mean IOP of both animals in DD was largely unaltered during subjective day and slightly reduced during subjective night relative to the entrained rhythm in LD. Figure 2B summarizes cosinor analysis of LD and DD data across animals (*n* = 10). Entrained and free-running rhythms did not significantly differ in IOP baseline (LD = 10.6 ± 2.5 mm Hg and DD = 10.1 ± 2.6 mm Hg, *P* = 0.56), peak-to-trough amplitude (LD = 10.9 ± 4.3 mm Hg and DD = 11.3 ± 4.5 mm Hg, *P* = 0.73), or period (LD = 24.0 ± 0.2 hours and DD = 24.1 ± 0.3 hours, *P* = 0.11). However, the free-running rhythm phase lagged by a small but significant amount (LD = −0.14 ± 0.50 hours and DD = 0.70 ± 0.58 hours, *P* < 0.01). The small lag is consistent with a free-running period close to 24 hours and with observations (data not shown) that it can take more than a week for the DD rhythm to drift noticeably out of phase with the external LD cycle.

**Figure 3.**
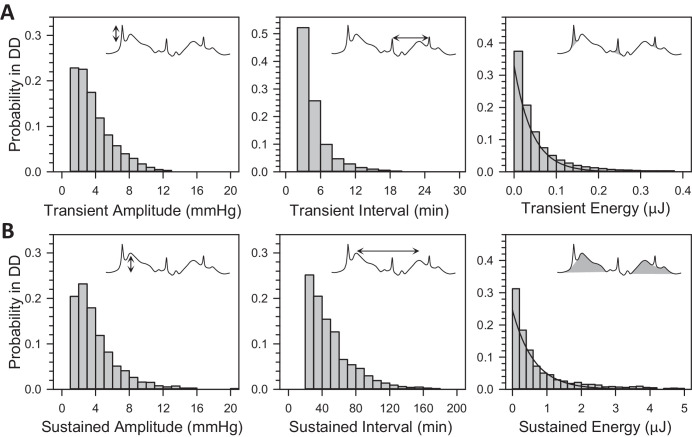
Statistics of rat IOP fluctuations in DD. (**A**) Probability distributions of transient event amplitude, interval, and energy across animals (*n* = 10). (**B**) Probability distributions of sustained event amplitude, interval, and energy across animals (*n* = 10). *Insets* depict amplitude (*left*), interevent interval (*middle*), energy content (*right*) of transient and sustained events (*shaded areas*). *Lines* indicate exponential fits of the transient and sustained energy distribution reported for rats in LD.^27^

Short-term fluctuations were analyzed to determine whether DD altered IOP statistics. The analysis parsed fluctuations into transient and sustained events of >2-minute and >20-minute intervals, respectively. Figure 3 plots the cumulative amplitude, interval, and energy distribution of these events across animals. The distributions were all nearly identical with those reported in LD.^27^ On average, there were 239 ± 61 transient events and 23 ± 5 sustained events per day in DD (*n* = 10). They are not measurably different from reported LD transient rates (231 ± 79, *P* = 0.80) but slightly less than reported LD sustained rates (29 ± 6, *P* < 0.05). The total energy content of transient and sustained events was 19 (16, 23) and 30 (23, 40) µJ per day, neither of which differed significantly from their reported energy content in LD (transient = 21 [19, 27] µJ/day, *P* = 0.53 and sustained = 33 [22,45] µJ/day, *P* = 0.54).

### Constant Light Cycle

**Figure 4.**
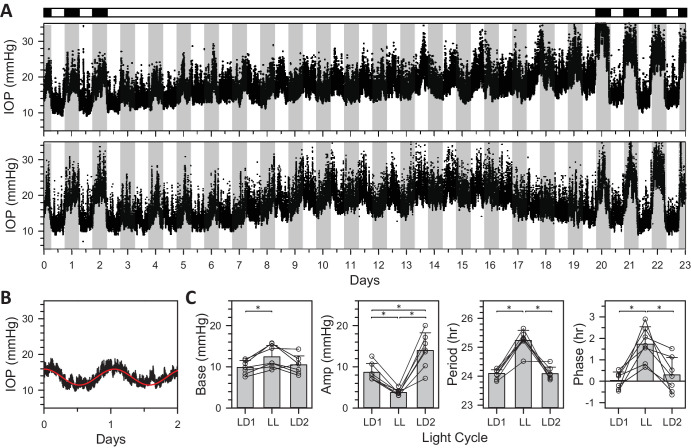
Effect of constant light on IOP rhythmicity. (**A**) IOP data recorded concurrently from two rats that were entrained to LD, exposed to LL for a few weeks, and returned to LD. *White and black horizontal bars*, respectively, indicate light and dark periods of ECU illumination experienced by the animals. *Gray vertical bars* indicate the dark phase of the ongoing LD cycle outside the ECU. (**B**) Average IOP across animals for the first 2 days in LL (*n* = 7). The *red line* is a single cosinor fit of the record. (**C**) Summary of rhythm properties in LL from cosinor analysis (*n* = 7). *Symbols* indicate base, amplitude, period, and phase estimates for individual animals in pre LD (LD1), LL, and post LD (LD2). Error bars give standard deviation. *Asterisks* indicate statistically significant differences.

Figure 4A shows IOP data collected simultaneously from 2 rats placed for a few weeks in LL. The IOP rhythm continued in LL for both animals but was markedly attenuated in amplitude. Baseline level drifted up with time, and rhythmicity eventually disappeared in most cases (*n* = 5 of 7). It is unknown whether all animals would have lost rhythmicity if kept longer in LL. Upon return to LD, the rhythm was immediately restored to normal or heightened amplitude. Figure 4B shows that LL waveform was also more sinusoidal and longer in period on average. As a result, the LL rhythm drifted over time and can be seen to peak around day 12 during daytime of the external LD cycle. Figure 4C summarizes cosinor analysis of LD and LL data across animals (*n* = 7). Baseline IOP of the free-running LL rhythm (12.4 ± 2.8 mm Hg) was higher than the entrained LD rhythm beforehand (9.9 ± 1.6 mm Hg, *P* < 0.05) and afterward (10.5 ± 2.2 mm Hg, *P* = 0.05), although the latter was not quite significant. In contrast, peak-to-trough amplitude of the LL rhythm (3.8 ± 0.8 mm Hg) was greatly reduced (pre LD = 8.7 ± 2.1 mm Hg, *P* < 0.001 and post LD = 14.0 ± 4.3 mm Hg, *P* < 0.01). The entrained LD amplitude was also significantly larger for multiple days afterward than beforehand (*P* < 0.01), suggesting that LL sensitizes the IOP rhythm to darkness. LL had a pronounced effect on the clock generating the rhythm because the free-running period was much longer than the entrained period (pre LD = 24.1 ± 0.2 hours, LL = 25.2 ± 0.4 hours; post LD = 24.1 ± 0.2 hours; *P* < 0.001 for LD-LL and *P* = 0.98 for LD-LD comparisons) and free-running phase was markedly delayed (pre LD = 0.0 ± 0.4 hours; LL = 1.7 ± 0.8 hours; post LD = 0.3 ± 0.8 hours; *P* < 0.01 for LD-LL and *P* = 0.74 for LD-LD comparisons).

**Figure 5.**
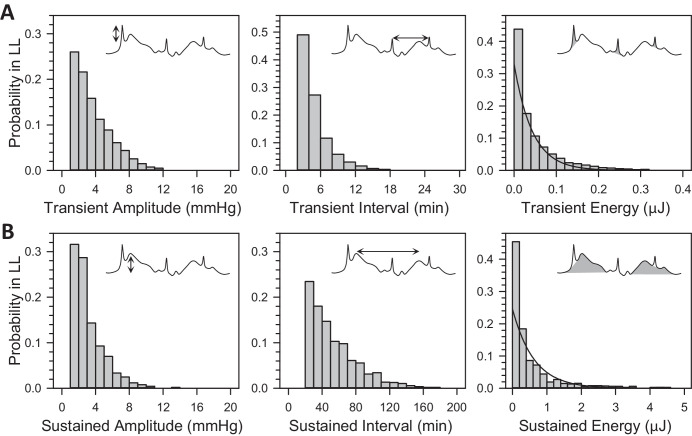
Statistics of rat IOP fluctuations in LL. (**A**) Probability distributions of transient event amplitude, interval, and energy across animals (*n* = 7). (**B**) Probability distributions of sustained event amplitude, interval, and energy across animals (*n* = 7). *Insets* depict amplitude (*left*), interevent interval (*middle*), energy content (*right*) of transient and sustained events (*shaded areas*). The *lines* indicate exponential fits of the transient and sustained energy distribution reported for rats in LD.^27^

Short-term fluctuations were analyzed to determine whether LL altered IOP statistics. Figure 5 plots the cumulative amplitude, interval, and energy distribution of transient and sustained events across animals (*n* = 7). The distributions were similar in shape to those in DD and LD, except for a relatively higher incidence of small events (<3 mm Hg). The excess of small events did not translate to larger total counts as there were 293 ± 27 transient events and 27 ± 4 sustained events per day on average, neither of which were significantly different from reported LD rates (transient: *P* = 0.06 and sustained: *P* = 0.40). However, the total energy content of transient (15 [13, 20] µJ/day) and sustained (17 [14, 23] µJ/day) events were both less than their reported energy content in LD (transient: *P* < 0.05 and sustained: *P* < 0.01), indicating that IOP variability was slightly reduced in LL.

### Reversed Illumination Cycle

**Figure 6.**
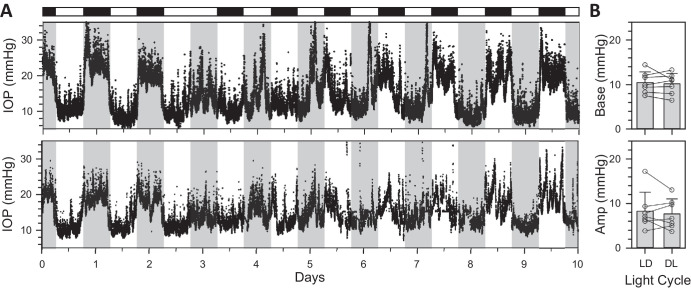
Effect of reversed lighting cycle on IOP rhythmicity. (**A**) IOP data recorded concurrently from two rats that were entrained to LD and then switched to DL. *White and black horizontal bars*, respectively, indicate light and dark periods of ECU illumination experienced by the animals. *Gray vertical bars* indicate the dark phase of the ongoing LD cycle outside the ECU. (**B**) Summary of rhythm properties in DL from cosinor analysis (*n* = 7). *Symbols* indicate base and amplitude estimates for individual animals. Error bars give standard deviation.

Figure 6A shows IOP data collected simultaneously from 2 rats placed for several days in DL. Much like LL, the rhythm of both animals was greatly diminished or abolished during the first subjective night by light. The rhythm waveform then appears to split into multiple slower peaks that merge over the following 4 to 6 days back into the step-like LD waveform, with IOP increases and decreases now temporally aligned to dark and light phases of the reversed illumination cycle and not the external LD cycle. All animals exhibited this pattern and time course of re-entrainment to DL (*n* = 7). Figure 6B summarizes cosinor analysis of LD and DL data, demonstrating that the IOP rhythm before and after re-entrainment was not noticeably different in baseline level (LD = 10.4 ± 2.4 mm Hg and DL = 10.2 ± 2.4 mm Hg, *P* = 0.65) or peak-to-trough amplitude (LD = 8.2 ± 4.3 mm Hg and DL = 7.7 ± 3.4 mm Hg, *P* = 0.54) across animals.

### Asymmetric Illumination Cycle

**Figure 7.**
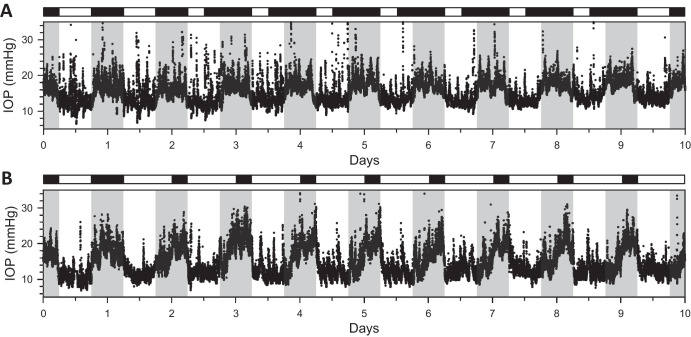
Effect of asymmetric lighting cycle on IOP rhythmicity. (**A**) IOP data recorded from a rat that was entrained to LD and then switched to 6L18D. (**B**) IOP data recorded from another rat that was entrained to LD and then switched to 18L6D. *White and black horizontal bars*, respectively, indicate light and dark periods of ECU illumination experienced by the animals. *Gray vertical bars* indicate the dark phase of the ongoing LD cycle outside the ECU.

IOP regulation by the circadian clock was perturbed with two asymmetric illumination patterns to probe clock sensitivity to photoperiod length, which varies seasonally. Figure 7A shows IOP data from a rat placed for several days in 6L18D. Shortening the photoperiod had no apparent impact on rhythmicity. IOP still peaked from 6 PM to 6 AM even though the subjective night commenced at noon in the ECU, and baseline and peak-to-trough amplitude were unchanged of this and other animals (*n* = 3). It is unknown whether the duration of IOP elevation would eventually match the dark phase if animals were kept longer in 6L18D. Figure 7B shows IOP data from another rat placed several days in 18L6D. Lengthening the photoperiod had no apparent impact on baseline and peak-to-trough amplitude on this or other animals (*n* = 3). However, it did progressively reduce the duration of the nocturnal elevation over 5 to 6 days to match the dark phase duration. The previous rhythm waveform returned immediately in LD (data not shown).

### Pulsed Illumination

**Figure 8.**
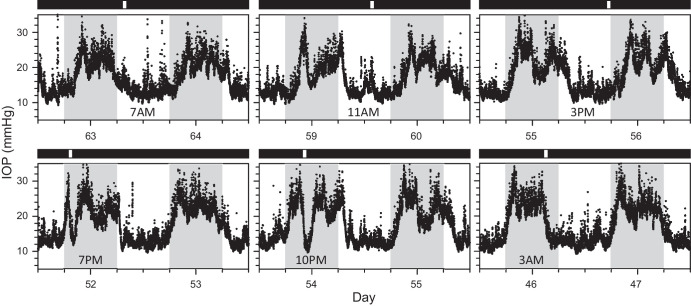
Effect of light pulses on IOP rhythmicity. IOP data recorded from a rat placed in DD on day 42 and subsequently exposed to 1 hour of light at different times during subjective day and night of their free-running DD rhythm (3 AM on day 46, 7 PM on day 51, 10 PM on day 53, 3 PM on day 55, 11 AM on day 59, and 7 AM on day 63). *White and black horizontal bars*, respectively, indicate light and dark periods of ECU illumination experienced by the animals. *Gray vertical bars* indicate the dark phase of the ongoing LD cycle outside the ECU.

Figure 8 shows IOP data from a rat in DD exposed at different times of day to a 1-hour light pulse. Light during subjective night elicited a large and immediate drop in IOP to the daytime level (time-to-trough = 64, 65, and 83 minutes, respectively, for 7 PM, 10 PM, and 3 AM pulses). IOP subsequently returned within 2 to 3 hours to the nighttime level if the pulse was after subjective dusk (7 PM or 10 PM), or it remained at the daytime level until the following evening if the pulse was before subjective dawn (3 AM). In contrast, light during subjective day had no apparent effect on IOP. All animals exhibited this pattern of nocturnal IOP suppression (*n* = 3). Light pulses at night also shifted rhythm phase on subsequent days. The phase response characteristic was not systematically investigated, but it underlies the delayed onset of nocturnal IOP elevation on days 56 to 64.

## Discussion

In this study, IOP rhythmicity was quantified under various ambient lighting conditions in free-moving rats using a wireless telemetry system that continuously collected data for several weeks without disturbing the animals. Rhythm waveform and response to light perturbations were captured with unprecedented accuracy, duration, and temporal detail. As previously documented by tonometry, rats housed under a standard LD cycle exhibit a diurnal IOP rhythm that peaks at night and troughs in the day. The rhythm is temporarily disrupted by surgical procedures associated with telemetry placement but recovers within a few days. The disruption may be related to the duration of anesthesia as the procedures can take a couple of hours and an attenuation of IOP rhythm amplitude was noted in mice after just 5 minutes of isoflurane inhalation.^30^ There is also growing evidence that general anesthesia perturbs circadian rhythms in sleep-wake behavior and many other biological variables.^31^^,^^32^

The diurnal IOP rhythm is driven by a circadian clock because it continued uninterrupted in DD. The source of rhythmicity is not certain as peripheral clocks have been identified in several ocular tissues of rodents, including the cornea,^33^^,^^34^ iris-ciliary body complex,^35^^,^^36^ and retina.^37^ However, the master driver of light entrainment is likely the central SCN clock through sympathetic and glucocorticoid pathways.^38^^,^^39^ The free-running period in DD was slightly but not significantly greater than 24 hours, meaning the IOP rhythm drifts little with time. Based on cosinor fits it would take almost 2 weeks to see a 1-hour phase lag with respect to LD. The finding is inconsistent with Aschoff's rules of photoperiodism, which state that the free-running DD period of nocturnal animals like rats should be <24 hours.^40^ The possibility that the clock entrained to another diurnal cue in the environment was discounted because the rhythm reversed in DL and remained so after several days in DD (data not shown). In addition, tonometry studies reported a phase advance of just 25 minutes per day for rabbits when the LD cycle was suddenly advanced 6 hours^25^ and similar IOP curves for rats in LD and DD,^6^ neither of which would be expected if the DD period differed markedly from 24 hours.

IOP rhythmicity is particularly sensitive to light at night. It was temporarily disrupted in DL until the clock re-entrained after several days to the reversed illumination cycle, and it was completely abolished for most rats in LL. The gradual dampening and eventual loss of rhythmicity in LL is consistent with tonometry studies.^7^^,^^9^^,^^17^^,^^41^ Those studies did not observe a concurrent increase in baseline IOP nor a lengthening of rhythm period in LL. However, a baseline increase in LL was seen by others.^42^^,^^43^ One study reported that the rat body temperature rhythm increased in the period by approximately 0.6 hours in LL, whereas IOP rhythm period was unchanged, which was interpreted as a decoupling of IOP and temperature clocks.^17^ This interpretation is likely mistaken due to sparse data given that IOP rhythm period lengthened by a comparable amount here. Moreover, Aschoff's rules of photoperiodism state that nocturnal animals should have a longer LL period.^40^

The effect of light at night is to block nocturnal IOP elevation. This was evidenced by IOP temporarily falling to the daytime level after 1-hour light pulses in DD and remaining at this level throughout the protracted light phase in 18L6D. Long photoperiods caused a similar shortening of locomotor activity rhythms in mice and sparrows.^44^^–^^46^ In contrast, darkness has no apparent impact on IOP rhythmicity. It proceeded in 6L18D unabated by the shorter photoperiod. Interestingly, light pulses have no impact either during the subjective day in DD, indicating that the inhibitory effect of light depends on the circadian time of day. The finding has potential implications for glaucoma studies that do not use telemetry to monitor IOP because brief exposure to light during hours of darkness, as could happen with routine animal care and non-sleeping patients, can disrupt rhythmicity and artificially lower tonometry readings.

Unlike IOP rhythmicity, IOP variability was not markedly influenced by ambient lighting conditions. The daily number, amplitude and interval distributions, and energy content of transient and sustained events were identical in LD, DD, and LL for the most part. A slight decrease in cumulative energy was detected in LL for both event types. This might reflect a general reduction in animal activity under constant illumination as transient events are associated with head and body motion.^27^ Aschoff's rules of photoperiodism also state that behavioral activity of nocturnal animals decreases with increasing light intensity.^40^ The decrease in sustained energy might reflect ECU isolation of animals given that sustained events have been attributed to startle, stress, and other autonomic processes,^27^ but then a comparable decrease would have been expected in DD. Alternatively, it might reflect heightened autonomic tone from the constant stress of LL on rats compared to more erratic activation in LD or DD. This is suggested by the steady increase in mean IOP and decrease in rhythm amplitude, both of which are consistent with reported effects of LL on rat blood pressure.^47^

Together, the results indicate that physiological mechanisms of the eye are intrinsically configured to maintain IOP at a basal daytime level. The action of the circadian clock is to alter these mechanisms each day so as to raise IOP from dusk to dawn. Light at night disturbs clock operation and its modulation of ocular fluid dynamics. Prior work indicates that the nocturnal IOP elevation is mediated by adrenergic signals that increase aqueous inflow and decrease aqueous outflow.^39^^,^^48^^,^^49^ The signals must target clock-specific pathways in the eye because light does not affect IOP during subjective day. It is important to elucidate these pathways and monitor their modulatory effects because nocturnal IOP correlates strongly with axonal injury in rat glaucoma models.^50^
